# Dengue Situation in Bangladesh: An Epidemiological Shift in terms of Morbidity and Mortality

**DOI:** 10.1155/2019/3516284

**Published:** 2019-03-10

**Authors:** Pulak Mutsuddy, Sanya Tahmina Jhora, Abul Khair Mohammad Shamsuzzaman, S. M. Golam Kaisar, Md Nasir Ahmed Khan

**Affiliations:** Communicable Disease Control (CDC), Disease Control Division, Directorate General of Health Services, Mohakhali, Dhaka 1212, Bangladesh

## Abstract

The escalating dengue situation in Bangladesh has been emerging as a serious public health problem in terms of morbidity and mortality. Results of analysis of 40,476 cases of Bangladesh occurring during 2000–2017 indicated that 49.73% of the dengue cases occurred during the monsoon season (May–August) and 49.22% during the post-monsoon season (September–December). However, data also showed that, since 2014, these trends have been changing, and dengue cases have been reported during the pre-monsoon season. During 2015–2017, in the pre-monsoon season, the dengue cases were reported to be more than seven times higher compared to the previous 14 years. The findings closely correlate with those of the pre-monsoon *Aedes* vector survey which revealed the presence of high density of larva and pupa of the dengue vectors in the environment all the year round. In our study, climate changes, such as average rainfall, humidity, and temperature, after 2014, and rapid unplanned urbanization were the strong predictors of an imbalance in the existing ecology that has led to increase in dengue cases in 2016 and the emergence of the chikungunya virus for the first time in Bangladesh in 2017. Although 2018 dengue data are relevant but not included in this study due to study time frame, it is interesting to report an increase in the number of dengue cases in pre (2016) and post (2018, which is highest within 18 years) chikungunya outbreak, which favors the study hypothesis. Despite the efforts to control dengue, based primarily on the vector control and case management, the burden and costs of the disease and similar vector-borne diseases will continue to grow in future in our country. Developing a cost-effective vaccine against all the 4 strains of dengue remains a challenge. The CDC, in collaboration with other research organizations, may come forward to initiate and coordinate a large-scale randomized clinical trial of an effective dengue vaccine in Bangladesh.

## 1. Background and Hypothesis

Although in recent years Bangladesh has achieved a remarkable progress in controlling communicable diseases, the country has still been facing a tremendous pressure in respect of public health problems especially controlling the emerging or re-emerging diseases. The upsurge in dengue cases and the recent outbreak of chikungunya and zika introduce major threats to the health of the community people. This emerging situation warrants an increased level of interventions and allocation of both human and financial resources in the health sector of Bangladesh. Bangladesh is situated in the tropical and sub-tropical regions like other Southeast Asian (SE) countries and like them has become a suitable habitat for the dengue vector and its increased transmission. Before 2000, only sporadic dengue cases were reported from Dhaka and other parts of the country [1, 2]. Dengue caused a serious public health concern, following a sudden outbreak in 2000 where around 5,551 cases and 93 deaths occurred in the country. During the dengue outbreaks from 2000–2017, both types of the vectors (*Aedes aegypti* and *Aedes albopictus*) were identified in Bangladesh [3, 4]. The re-emergence of dengue and the recent emergence of the disease due to chikungunya viruses, both spread by the *Aedes* mosquitoes, are very worrying and have created a huge burden in morbidity and mortality with insufficient allocation of resources under the CDC Operational Plan (OP) of the Health, Population, and Nutrition Sector Program (HPNSP: 2017–2022) [5, 6]. Like other countries, once the virus have emerged in Bangladesh, they are expected to remain in the environment as the vector is always present and will cause increased public health problems in the near future.

**Figure 1:**
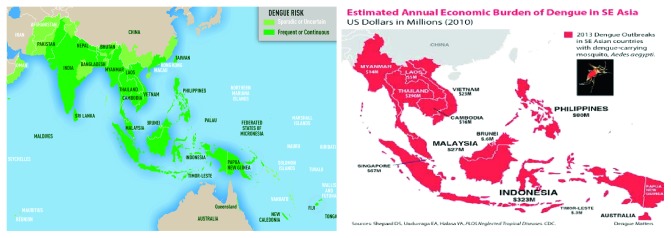
Map showing the countries where the population is at risk due to dengue, in particular Bangladesh, and also showing an estimated economic burden in the Southeast (SE) Asia region, where the economic burden is growing specifically in Bangladesh. Source: CDC, Atlanta, 2010 [7].

A glimpse of the dengue situation in Southeast Asia can provide a picture of how this emerging disease is causing a huge economic and social burden in Southeast Asia, in particular, and Bangladesh, in specific [7, 8]. Like other lower and middle income countries (LMICs) as shown in Figure 1, the existing dengue situation in Bangladesh is creating economic burden in our health sector as the allocation of health budget is gradually declining by year and, at the same time, the out-of-pocket expenditures (OOPs) are increasing (67%, which is highest in the Southeast Asia region) according to the study findings of Bangladesh National Health Accounts (BNHA-V) [9].

The Health Sector Plan in Bangladesh, 4^th^ Health, Population and Nutrition Sector Program (HPNSP: 2017–2022) [10], aims to ensure a quality and equitable health care for the entire population by improving their access to assured quality of care, particularly by the poor, women, and children. Under this Sector Program, the Ministry of Health and Family Welfare intends to establish a people-oriented responsive health care system to achieve health-related goals to contribute to the “Sustainable Development Goals” (SDGs) by 2030. Altogether the HPNSP has 32 operational plans of which Communicable Disease Control (CDC) is important, it has gained attention due to emerging and re-emerging diseases in recent years.

CDC is one of the functional units of the Directorate General of Health Services (DGHS) under the Ministry of Health and Family Welfare (MoHFW) of Bangladesh. Until 1999, the activities of CDC were attached to the Directorate of Primary Health Care (PHC) and were delivered by a Deputy Director, CDC. Following the serious outbreak of dengue in Dhaka city in 2000, the Ministry of Health and Family Welfare established CDC as an independent unit under a separate division, Disease Control Division in the DGHS to cope with the emergency management crisis of the dengue situation. As a Disease Focal Point, the CDC was established to keep the communicable disease situations under control throughout country, so that it can never be a serious public health problem for the people of Bangladesh. Currently, the CDC has seven components under its Operational Plan (OP) in the HPNSP, where *Aedes* mosquito-borne diseases such as dengue, chikungunya, and zika remain as sub-components under the Malaria Elimination Control Program [11].

## 2. Hypothesis and Objective of the Study

The *Aedes* mosquitoes are responsible for the transmission of dengue, chikungunya, and zika in our country. This vector is mainly a domestic habitat, highly climate sensitive in the environment, and breeds in a small quantity of water especially in urban environments.

The studies described in this paper seek to answer the following question: “After 2014, what ecological imbalances occurred in the existing environment due to the change in climate factors (rainfall, humidity, and temperature) and possible rapid unplanned urbanization which caused a sudden shift in the epidemiological characteristics of dengue causing a large upsurge in cases and deaths in 2016 and contributed to the emergence of a chikungunya outbreak for the first time in Bangladesh during 2017 and observing an increased reporting of cases in the pre-monsoon season?”

## 3. Methods and Materials

The CDC usually monitors the communicable diseases situation throughout the country by collecting information through its control room cell. The Control Room of the Directorate General of Health Services was established in 1983 with the technical assistance of International Centre for Diarrhoeal Disease Research, Bangladesh (icddr,b), under a collaborative program, The Epidemic Control Preparedness Program (ECPP) with the Ministry of Health and Family Welfare, Bangladesh. The control room operates throughout 24 hours and collects information mainly on dengue, diarrhea and other health related emergencies. The control room cell is now designated as the National Health Crisis Management Centre (NHCMC), DGHS.

### 3.1. Data Collection

#### 3.1.1. Routine Data on Morbidity and Mortality

Dengue surveillance is mostly conducted actively either by communicating directly with the public and private health facilities or passively through “Hot Line” daily. Besides, at the end of the month, each facility produces a cumulative report of morbidity and mortality on the dengue situation and submits it to the CDC. In addition, information on treatment seeking pattern on dengue cases from the public and private hospitals were recorded for the last five years (2013–2017). Although data were available from 2000, the study principles tried to concentrate our analysis on the last five years to avoid any information bias. As death is always a crucial indicator to design program-specific interventions, the study team conducted verbal autopsy as retrospective analysis of deaths due to dengue to gather information on duration of illness, cause of deaths, etc. within the last five years (2013–2017) to avoid any recall bias. Due to unavailability of their locations at the admission period, in most cases we limited our analysis to clinical record review at the facility level.

#### 3.1.2. *Aedes* Mosquito Survey Data

*Aedes* mosquito survey data were collected from Dhaka city from 2013 onwards. The aim of the survey was to determine the spatial distribution of dengue vectors in Dhaka city and to use the results at the policy level for action planning. The CDC, in collaboration with the Zoology Department of the Jahangirnagar University and with the financial assistance from the World Health Organization (WHO), Bangladesh, conducted the *Aedes* survey in Dhaka city. As Dhaka city is a big area with rapid unplanned urbanization with 50 zones and 93 wards with a huge population and a large number of households, the survey was carried out using a 30-cluster sampling techniques, as recommended by the WHO and as used for evaluating the Expanded Programme of Immunization (EPI) coverage in Bangladesh [12]. The environmental samples were collected in each household unit within the sampling framework. All the possible habitats of the dengue vectors were explored within the sampled households and their immediate surroundings environment.

#### 3.1.3. Knowledge, Attitude, and Practice (KAP) Survey Data

Knowledge, attitude, and practice (KAP) survey data on dengue and chikungunya were collected during the pre-monsoon *Aedes* survey period of 2016. A structured pre-tested closed questionnaire in English and local Bangla language was used, and the head of the family members of each sampled household unit was interviewed for KAP information on dengue and disease morbidity. A circular was given to select the interviewers who had the previous experiences of similar field work among the students of the Entomology and Zoology Department of the Jahangirnagar University, and the selected students were trained on the survey instruments. The total sample for both the entomological and KAP survey was *n*=997. The KAP survey was conducted in conjunction with the entomological *Aedes* survey in the pre-monsoon period, 2016.

#### 3.1.4. Climate Data

The climate data relating to rainfall, humidity, and temperature in terms of maximum, average, and minimum, for the 2015–2017 periods were collected from the Metrological Department of Dhaka, Bangladesh.

Since the CDC has no capacity or funds to conduct virology testing, it has to depend on the tertiary laboratory facilities. So, there is no routine yearly laboratory surveillance for dengue sero-typing. In 2013, the CDC, in collaboration with the Virology Department of Bangabandhu Sheikh Mujib Medical University (BSMMU), conducted studies to determine the sero-type circulating in that period only with the financial assistance from the WHO, Bangladesh.

#### 3.1.5. Data Analysis Plan

The data-analysis plan was simple and included running frequency tables and some cross tables. The SPSS software (version: 20) was used for analyzing data. No advanced analysis was planned due to the nature of the data generated from the daily dengue surveillance of the HMIS (Health Management Information System), which was based only on morbidity and mortality information. For the KAP survey data, a limited significance test (chi-square) was applied. The monthly climate data were correlated (Spearman correlation, 2-tailed) with the monthly reported confirmed dengue cases from the 2014–2017 period. In the entomological survey, the main vector indexes (Household Index, Breteau Index, and Pupa Index) were used for determining the vector density, and Geographical Information System (GIS) was used for showing the spatial distribution of vectors based on the entomological survey results.

## 4. Results

Results of the study are presented under *four headings*: Morbidity and Mortality Pattern, Seasonal Variation Affecting Dengue Cases and Deaths, Climate Factors in Terms of Disease Incidence, and finally, the *Aedes* survey results to establish the vector density with seasonal variation.

### 4.1. Morbidity and Mortality Patterns, 2000–2017

**Figure 2:**
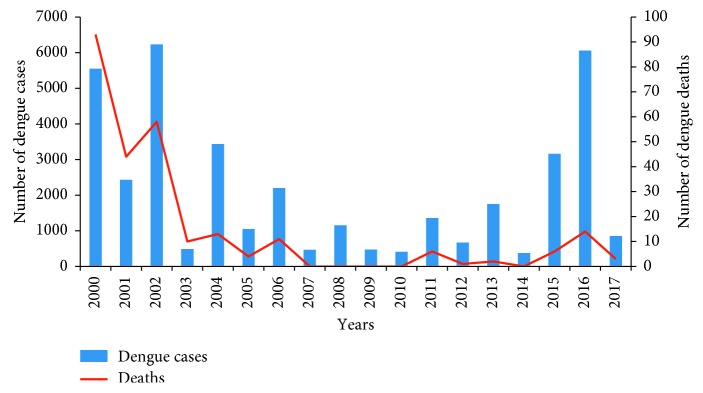
Trend analysis of reported dengue cases and deaths by years from public and private health facilities by the Control Room, DGHS, 2000–2017, is shown. It also shows the maximum number of deaths was in 2000, when dengue emerged as the first outbreak in Bangladesh, and the study analysis earmarked the period as the pre-preparation phase. The map also shows a sudden rise of dengue cases in 2016 and subsidence of dengue cases in 2017 but leads an explosion of the chikungunya outbreak for the first time in Bangladesh, which reflects the study hypothesis.

The trends of dengue situation in Bangladesh were analyzed from 2000 to 2017 (Figure 2). In total, 40,476 confirmed dengue cases were reported. Two peaks were demonstrated in 2002 and 2016. The maximum number of deaths occurred in 2000, when the dengue cases first emerged in an epidemic form in Bangladesh.

For analysis purpose, we divided the whole period into five years intervals because resource allocation under the reform process of sector-wide approaches (SWAps) of the Ministry of Health and Family Welfare was started in 1998 under the Health, Population Sector Program ((HPSP: 1998–2002), as per the Operation Plan (OP) for five years. So, we earmarked the whole period in two stages: Pre-preparation phase (1998–2002) and Intervention phase (2003–2017). The main purpose was to address deaths due to dengue and to formulate a policy for action. Although the CDC operated their activities since 2000, some activities were still planned in the Primary Health Care (PHC) Operational Plan. The intervention phase is a long-term effort, where lots of activities were accomplished as planned in the Operational Plan for capacity building of the Ministry of Health and Family Welfare and the Ministry of Local Government, Rural Development and Co-Operatives (MoLGRD). These activities are often overlapping, and their impact is difficult to separate.

In the pre-preparation phase illustrated in Table 1, there are a large number of cases and more deaths with a case fatality ratio (CFR) of 1.38%, which is higher than the acceptable range. Seventy-three percent (73%) of the total deaths occurred in this phase. Due to subsequent innovative actions taken by the CDC during the intervention phase, a CFR below 1% (0.65%) was achieved.

#### 4.1.1. Morbidity Analysis

The morbidity analysis in terms of health seeking behavior in the last 5 years (2013–2017) showed 14,121 cases and 28 deaths (Table 2). The private health facilities treated 78% of the total cases, but the CFR is around 4 times less than that of the public facilities. Although the public health facilities treated 22% of the total cases, the CFR was much higher. Some assumptions of the findings are highlighted in Discussion, which is difficult to explain and as such, it needs in-depth investigation and appropriate action to improve the quality of care in the public sector.

#### 4.1.2. Dengue Mortality Analysis

We tried to minimize recall bias. The five-year (2013–2017) clinical records of deaths in both public and private health facilities were reviewed. The majority of deaths were located in Dhaka city (93%) and were males (68%) and within the 15–50 year old age groups (57%). The main causes of death (65%) were complications due to dengue: DHF (29%; dengue hemorrhagic fever) and DSS (36%; dengue shock syndrome). The remaining number of deaths was diagnosed as dengue fever (36%; DF). More than 70% of the deaths occurred within 24 hours of admission to the hospital (Table 3). This indicates the need for improved clinical management of dengue complications at the facility level and an emphasis on seeking an early treatment by dengue patients. Although relevant information of 2018 was not included in this study, deaths due to dengue were reported in the early part of the pre-monsoon season and highest number of dengue cases (10,148 cases and 26 deaths) were reported within 18 years, which is a new trend in Bangladesh (Source: Control Room, DGHS).

### 4.2. Seasonal Relationship

**Figure 3:**
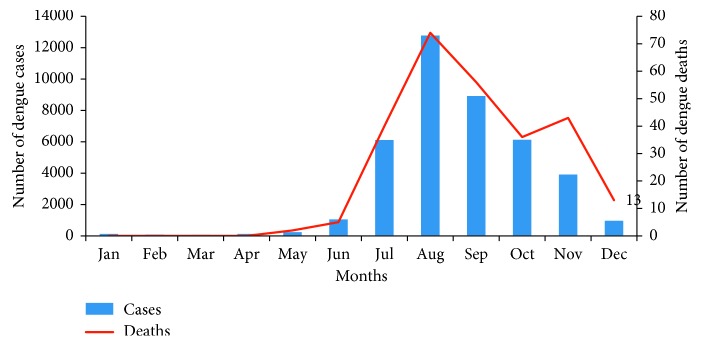
Seasonal variation in the occurrence of dengue cases and deaths by month, 2000–2017, shows less cases in the pre-monsoon season (January–April), and maximum cases and deaths were reported in the monsoon season (May–August) and in the post-monsoon period (September–December). Figure 3 also shows the peak season of dengue in Bangladesh is from July to October.

Considering seasonal relationship, as the dengue cases in Bangladesh have a close relationship with seasonal variation, we classified the whole period within a narrower margin (3 years interval). Of the 40,476 cases reported from 2000 to 2017 (Table 4), less than 1% (0.94%) were in the pre-monsoon season. The dengue cases were mostly reported in the monsoon period (50%) and in the post-monsoon season (49%), and the peak season of dengue in Bangladesh is from July to October (Figure 3). The situation changed suddenly after 2014, when more dengue cases were reported in the early part of the year. Within the last three years (2015–2017), the dengue cases reported in the pre-monsoon season were seven times higher than that of the previous 14 years. More dengue cases were also reported in the post-monsoon period, which is unusual. This may be due to intermittent rainfall, early onset of the monsoon, and prolongation of the monsoon season throughout the year. This warrants more focused attention on the environmental aspects associated with dengue transmission, as during 2017, the number of dengue cases was reported comparably less throughout the year, but there emerged a huge outbreak of chikungunya virus for the first time in Bangladesh in Dhaka and other parts of the city.

### 4.3. Climate Variation

**Figure 4:**
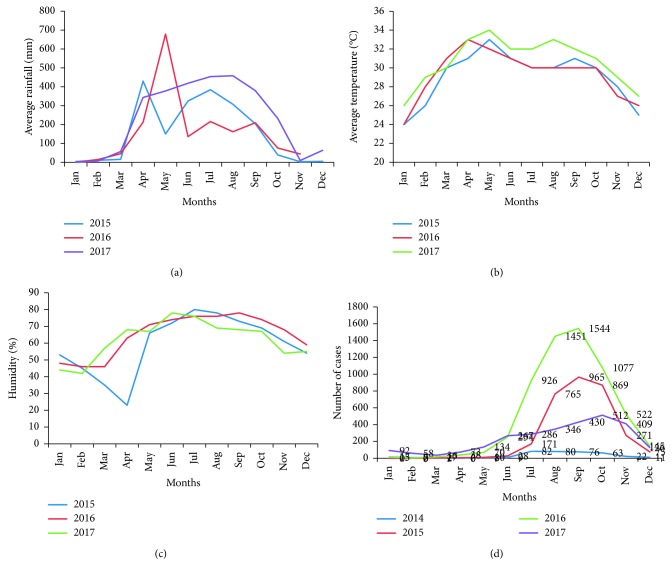
Average climate data (rainfall, temperature, and humidity) after 2014 show correlation analysis with the dengue cases from 2014 to 2017. The analysis showed a significant relationship between dengue incidence and climate factors (*p* < 0.001 with humidity, *p* < 0.019 with temperature, and *p* < 0.039 with rainfall): (a) comparison of rainfall by year, 2015–2017; (b) comparison of temperature by year, 2015–2017; (c) comparison of humidity by year, 2015–2017; (d) comparison of dengue cases by year, 2014–2017.

Climate variation (rain fall, humidity, and temperature) formed part of the analysis of dengue fever trends as the dengue vector is very sensitive to climate change and the resulting environmental modifications. Since there is, to some extent, linear relationship between climate factors and the mutation process of the organisms (virus or bacteria) [13], we tried to correlate the climate information (Figures 4(a)–4(c)) with the incidence of disease from 2014 (Figure 4(d)). The study showed a strong and significant correlation with humidity and positive dengue cases (*p* < 0.001) and also showed a significant correlation with low and medium rainfall (*p* < 0.039). The results of one-way ANOVA and multiple regression analysis also showed a significant relationship with positive dengue cases and the average temperature (*p* < 0.019). In addition, the study sought to explore the underlying relationship between sudden onset of chikungunya outbreak and the climate factors in the existing ecology. Results of analysis of the climate information showed that the average rainfall, humidity, and temperature were comparatively higher in the last three years (2015–2017) than that of the previous years.  Figure 4 illustrates that, with the variation of the climate, the dengue cases also flared up, and there was an upsurge in 2016. This ecological change probably promoted the mutation process among the viruses in the environment and was accompanied by the emergence of chikungunya virus for the first time in Bangladesh. McCarty et al. reported “Climate change may benefit some species and cause extinction for others. Cumulatively, it will alter biological communities and the functioning of ecosystems” [13]. Results of similar studies also suggest a linear relationship with the incidence of dengue due to climate variations in Bangladesh by Karim et al. [14]. Similar findings due to climate impact were observed by Epstein [15], and Gould and Higgs [16].

### 4.4. Entomological Studies

Since entomological studies are very important in controlling vector-borne diseases, the CDC, since 2013, started to conduct entomological studies to determine the vector concentration in Dhaka city. Surveys were mainly conducted in the pre-monsoon period; however, from 2017 onwards, monsoon and post-monsoon *Aedes* survey were planned to conduct every year to determine the density, distribution, and breeding habitats of the *Aedes* vector(s) and to plot spatial mapping based on the larvae and pupae density. Results showed the variable spatial distribution and density of *Aedes aegypti* pupae and larvae were spread across many areas and were relatively high in Dhaka city.

**Figure 5:**
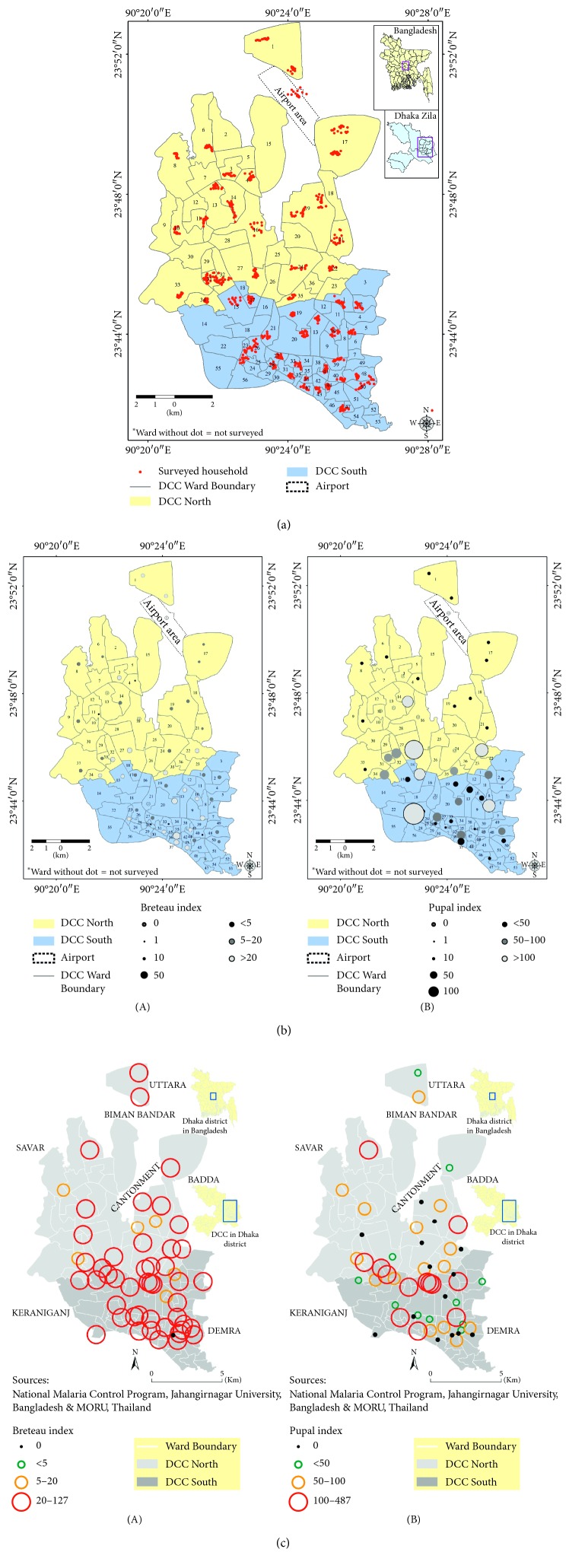
Map showing the spatial distribution of surveyed households for *Aedes* and KAP study in Dhaka city areas (north and south city corporations) during the pre-monsoon and monsoon period, 2016 and 2017 (a), and spatial distribution of dengue larvae and pupae (b) in the surveyed areas of Dhaka city, and density of chikungunya vector(s) mosquitoes in Dhaka city corporations (North and South) during the monsoon period, 2017 (c), when dengue cases were mostly suppressed and an outbreak of chikungunya was reported for the first time in Bangladesh. The map also shows higher ranges of Breteau Index (BI) and Pupa Index (PI) in most of the surveyed areas. (a) Spatial distribution of surveyed household in Dhaka city corporation areas during the pre-monsoon period. (b) Density of dengue vector(s) both in larvae and pupal stages in Dhaka city during the pre-monsoon period. (c) Density of chikungunya vector(s) in Dhaka city corporations, 2017.

*The results of pre-monsoon Aedes survey* in 2016 showed that the most common outdoor habitats of *Aedes* vectors were plastic buckets (13%), plastic drum (11%), and clay pots (7%); the most productive wet indoor habitats of *Aedes* vectors were plastic drums (15%), buckets (15%), flower tubs and trays (2%), and water tanks (0.77%) (Table 5). Other habitats consist of a large variety of indoor and outdoor containers where clear water accumulates. Results of the subsequent survey in 2017 revealed that, in the monsoon and post-monsoon periods, the concentration of vectors is much higher (Figures 5(b) and 5(c)). We may conclude from the findings of our survey that the dengue vectors are present in the domestic and outdoor environment throughout the year in Bangladesh. At the same time, the results of a KAP survey among the city dwellers within the sample framework revealed that most (51%) city dwellers do not know the breeding habitats of *Aedes* mosquitoes but have a good knowledge (61%) of the daytime biting habit of *Aedes* mosquitoes [17–19].

## 5. Discussion

The CDC is a service delivery unit of the Directorate General of Health Services (DGHS) under the Ministry of Health and Family Welfare (MoHFW). Its mission is to control communicable diseases in the country; it is not a research organization. The CDC is within the organizational structure of the DGHS and implements the programs, and it is overseen by the Ministry of Health and Family Welfare. Due to such limitations, a structured study design is not provided in this paper. The data generated here are a by-product of the intervention process at the field level. It includes routine surveillance data collected by the control room, DGHS, and the monitoring data collected at the field level, which provides only a limited scope for advanced analysis. The daily surveillance data used in this study are based on morbidity and mortality information on dengue cases throughout the country. The very nature of such a data source will entail questions about the issue of validity and quality of data, which is a clear limitation of this study. Despite this data limitation, the information generated by the analysis has important implications at the policy level and leads to appropriate action being taken against the emerging diseases such as dengue and chikungunya, which are now the major public health problems in Bangladesh.

Results of analysis of 40,476 confirmed dengue cases showed that, in the pre-preparation phase (1998–2002), most lives lost due to sudden exposure to the dengue virus for the first time in Bangladesh. This was due to lack of proper management of the dengue cases in terms of lack of assessment of the needed resources, trained man power, coordination, diagnostic and treatment facilities, and overall lack of awareness among the population and the medical providers. In subsequent years which we termed the intervention phase (2003–2017), the Ministry of Health and Family Welfare and the Ministry of Local Government, Rural Development and Co-Operatives (MoLGRD) took on-board lessons learnt and developed capabilities to keep this emerging dengue situation under control successfully. Interventions needed were identified for implementation, including training of human resources, development of clinical guidelines, establishment of treatment facilities, ensuring good referral linkages, procurement of Rapid Diagnostic Kits, promotion of an Integrated Vector Management (IVM) strategy, and intensive ACSM (Advocacy, Communication and Social Mobilization) activities for community involvement and development of a Country Action Plan as a part of prevention and control activities. The combination of all these activities reduced the case fatality ratio to below 1% [20–22]. This very satisfactory result was due to a combination of long-term efforts and interventions. It is therefore very difficult to assess the impact of any individual intervention.

Most cases were reported from the private facilities as the numbers of private facilities are located in urban areas. The reason behind the preferred private facilities may be explained by the people's perception of public facilities. People may perceive that there are additional hazards and barriers when seeking admission and treatment from the public facilities. Results of a study by Mutsuddy et al. showed that the use of public facilities varied by the economic status where poor and lower-middle income patients having a general tendency to visit the public health facilities, but more valuable investigations and treatments available in the public sector are used by the upper class group [23, 24]. As we do not have the socio-economic information of the dengue patients, we may assume that most patients who were visiting the private facilities are of a better socio-economic status. We have identified a major concern that the public hospitals treat fewer cases but have more deaths, despite the fact that the public hospitals have more resources in terms of trained man power and good patient-monitoring systems. However, the public facilities may deal more complicated cases, skewing their data on case fatalities. By nature as profit-making bodies, there is a tendency in the private facilities to avoid the occurrence of any deaths to maintain their reputation, so they refer more complicated cases to the public facilities to minimize the impact on their reputation. This is a burning issue that needs to be resolved and requires a further study to improve the utilization and reputation of the public facilities in the country.

Of all the deaths reported within the last five years (2013–2017), more than 70% occurred within 24 hours. This is alarming in terms of case management of dengue-related complications, such as DHF or DSS, and the correct implementation of the disease-monitoring system. In most dengue cases, body temperature usually subsides within 3–5 days and patients are released from hospitals, if admitted. During the stay at home, the patient may develop symptoms associated with severe dengue, such as hemorrhage, abdominal pain, and shortness of breath as complications [25–27]. The results of our study revealed that 65% of the deaths were due to complications, such as DSS or DHF.

In Sri Lanka, Rajapakse observed associated co-morbidity among the dengue shock cases as a complication [28]. The present study could not confirm such an issue due to limited access to information. The deaths due to dengue in Bangladesh are associated with many factors from both the patient and provider perspective. Late decision for hospitalization when complications are far advanced is one of them, when there is very little left to do to prevent death. Lack of the provider's skill to handle DHF or DSS may be another important factor. Bangladesh has a little scope to deal with DSF/DSS cases compared to Sri Lanka, where the providers are more equipped in both expertise and logistics support to deal with DHF and DSS cases with subsequent minimum casualties [29]. These issues, from both demand and supply side, contributing to mortality, need to be addressed to reduce mortality from complications due to dengue in Bangladesh.

The incidence of dengue cases in Bangladesh show a marked seasonal variation, although this is becoming less marked in recent years. This study has shown that most dengue cases occur in the monsoon and post-monsoon season. Similar results of seasonal variation in dengue cases in Dhaka city were reported by Karim et al. and Morales et al. [14, 30]. The season-specific concentration of dengue cases was well reported in many other similar studies in Southeast Asia and other countries [31–33]. The findings of our study on seasonal variation also matched in the adjacent geographical locations of Myanmar and India [34, 35]. In the pre-monsoon season for the last 14 years (2000–2014), a negligible number of cases were reported; however, since 2015, the dengue cases started to be reported from the early part of the year, and within the last three years (2015–2017), the number of dengue cases reported in the pre-monsoon season is seven times higher than those reported in the previous 14 years. In addition, in the early part of 2018, a dengue death occurred in the pre-monsoon season for the first time in Bangladesh, and highest number of cases (10,148; although very relevant but not included in the study due to study time frame) were reported within last 18 years (Source: Control Room, DGHS), which is also in support of the study hypothesis. These findings both in terms of dengue morbidity and mortality are the evidence of a new epidemiological trend in Bangladesh, and surprisingly in 2017, we identified an outbreak of chikungunya for the first time in Bangladesh. So, we may assume that climate changes in rainfall, humidity, and temperature since 2014 have caused an ecological imbalance in the environment that has resulted in a major shift in disease incidence during the pre-monsoon season and facilitated the introduction of transmission of the chikungunya virus in the country. This effect of climate change was observed by Siddque et al. during the 1991 cholera outbreak in Bangladesh, when classical *Vibrio cholerae 01* was completely replaced by *Vibrio cholerae 0139 Bengal* due to ecological changes in the Bay of Bengal [36].

A small-scale laboratory surveillance by the IEDCR (Institute of Epidemiology, Disease Control and Research) reported 13,814 chikungunya cases (Source: Director, Disease Control), which is more than four times higher than the dengue incidence in 2017. As the clinical presentation of chikungunya is almost similar to dengue and many other viral diseases, it was very difficult to differentiate for case reporting. As the disease emerged for the first time in Bangladesh, people had no prior immunity against the disease: so, as in many other countries, it flared up very quickly, and almost every household was affected. In reality, the chikungunya cases may have been many more than were recorded. The average rainfall, temperature, and humidity were comparably higher since 2015 and onwards as demonstrated in the Figures 4(a)–4(c); we may assume an increasing imbalance in the ecology, which increases the likelihood of the emergence of the chikungunya virus and possibly other viruses such as zika for the first time in Bangladesh. From the study analysis, it reveals the increase in average and minimum rainfall which significantly correlated with an increase in dengue cases. Our findings corroborate the findings of other similar studies in many other countries in the same geographical regions [37, 38]. At the same time, our data showed that the rise of average temperature was also linked to increased dengue infection. It is known that temperature increases lead to expansion of the area involved and the number of cases of vector-borne diseases [39, 40]. Similarly, the present study showed a highly significant correlation with humidity and the increase in dengue cases. These conditions facilitate the growth and survival of mosquitoes needed for successful propagation of the virus in the environment [41, 42]. The impact of climate on an increased dengue case reporting is suggested by other similar studies [43, 44]. Simultaneously, results of the entomological studies, carried out by the CDC in 2017, suggested a high percentage of Breteau Index (BI) and Pupal Index (PI) of the *Aedes* vector both in terms of larvae and pupa numbers in Dhaka city throughout the year.

The spatial distribution that denoted the density of *Aedes aegypti* and the larval indices, which were relatively high in Dhaka city, motivated all the stakeholders to take immediate action as in other countries [45]. In our *Aedes* survey findings, the important reservoirs of vectors are almost same as in other Southeast Asian countries [46, 47]. It would be very interesting to know whether, during the whole period of 2017, the vector *Aedes* mosquitoes carried either dengue or chikungunya virus or both. Chen et al. found both dengue and chikungunya infection at the same time among travelers [48, 49]. This was a missed opportunity by the CDC to explore and a need to prepare for subsequent actions.

## 6. Conclusion

This study concentrated only on the epidemiological aspects of dengue-related morbidity and mortality in terms of climate factors and seasonal variation. It has also demonstrated a shift in the occurrence of dengue incidence as more dengue cases are being reported during the pre-monsoon season due to climate change and possibly other factors such as rapid unplanned urbanization and other urban parameters. From the epidemiological and entomological perspectives, the results of our study predict that the climate changes after 2014 may have caused some ecological imbalances in the environment that leads to occurrence of more dengue cases in the pre-monsoon season and has also led to an explosion of chikungunya for the first time in Bangladesh in 2017. Although 2018 dengue data are relevant but not included in the study due to study time frame, it is interesting to report an increase in the number of dengue cases in pre (2016) and post (2018, which is highest within 18 years) chikungunya outbreak, which are also in favor of the study hypothesis. Further studies are needed to explore with other environmental variables, serological prevalence of dengue virus and clinical and immunological data.

Dengue, an emerging disease, will remain in Bangladesh and will continue to constitute a serious public health problem as is happening worldwide. The changing epidemiology should be clearly understood, and constant monitoring is needed, including extending the surveillance areas and addressing the challenges to reduce the impact of the disease on public health and the economy of the country. It may be very challenging to root up the disease from the supply side, and a long-term investment is needed to achieve behavioral changes in the urban population to join the fight against the *Aedes* mosquitoes. The CDC has conducted only one sero-type study long before 2013; the predominant strain was DEN-2 (87%) [50]; on further study, it was revealed that DEN-1 gained predominance at the end of 2016 in Dhaka city (Personal communication: Chairman, Virology Department, Bangabandhu Sheikh Mujib Medical University (BSMMU). In fact, all the four sero-types of the dengue virus are now existing in the country. There is at present only one licensed vaccine (Dengvaxia from Sanofi) in the market, which is limited to population aged over nine years of age and has only about 65% efficacy [51]. The CDC, in collaboration with other research organizations as in India [52], may come forward to initiate and coordinate a large-scale randomized clinical trial of this dengue vaccine in Bangladesh.

## Figures and Tables

**Table 1:** Case fatality ratio before and after the intervention by the operational plan, 1998–2017.

Phase	Cases	Deaths	CFR (%)
Pre-preparation phase			
1998–2002	14,213	195	1.38

Intervention phase			
2003–2007	8530	38	0.44
2008–2012	4014	07	0.17
2013–2017	14,121	28	0.19

Sub-total (intervention phase)	26263	173	0.65
Total (2000–2017)	40,476	268	0.66

CFR = case fatality ratio.

**Table 2:** Treatment seeking pattern of dengue cases and deaths, 2013–2017.

Health facilities	Cases	Deaths	CFR (%)
Public facilities	3304 (22%)	14	0.42
Private facilities	10,817 (78%)	14	0.12
Total (2013–2017)	14,121 (100%)	28	0.19

CFR = case fatality ratio.

**Table 3:** Duration of death after hospitalization and causes of deaths due to dengue, 2013–2017.

Duration of death after hospitalization	No. of deaths (%)	DHF	DSS	DF
<24 hours	20 (71.4)	5	9	6
1–5 days	3(10.7)	1	0	2
More than 5 days	5 (17.9)	2	1	2
Total	28 (100)	8 (28.57%)	10 (35.71%)	10 (35.71)

DHF = dengue hemorrhagic fever; DSS = dengue shock syndrome; DF = dengue fever.

**Table 4:** Seasonal variations of dengue cases: a comparison by year.

Year	Pre-monsoon	Monsoon	Post-monsoon	Total
2000–2002	0	7193	7020	14213
2003–2005	4	2802	2703	5509
2006–2008	0	2985	834	3819
2009–2011	0	1305	885	2190
2012–2014	43	1174	1587	2795
2015–2017	334	4671	6945	11950
Total (2000–2017)	381 (0.94%)	20130 (49.73)	19224 (49.22%)	40476

**Table 5:** Most productive container for vector breeding places by location.

Location	Type of a wet container	No. of larva pupae collected	*Aedes aegypti* (%)	*Aedes albopictus* (%)
Indoor	Plastic drum	352	15.27	0
	Plastic bucket	307	14.75	0
	Flower tab and tray	99	2.4	0
	Tires	196	5.76	2.69
	Water tank	28	0.77	0

Outdoor	Plastic bucket	307	12.65	0.13
	Plastic drum	171	11.08	0
	Clay pot	146	7.24	0
	Tires	135	7.17	0
	Water tank	136	6	0

## Data Availability

The data used to support the findings of this study are available from the corresponding author upon request.
